# Editorial: Wild plant genetic resources: a hope for tomorrow

**DOI:** 10.3389/fpls.2023.1217547

**Published:** 2023-05-31

**Authors:** Mohd. Kamran Khan, Tofazzal Islam, Sait Gezgin, Francesco Di Gioia

**Affiliations:** ^1^ Department of Soil Science and Plant Nutrition, Faculty of Agriculture, Selcuk University, Konya, Türkiye; ^2^ Institute of Biotechnology and Genetic Engineering, Bangabandhu Sheikh Mujibur Rahman Agricultural University, Gazipur, Bangladesh; ^3^ Department of Plant Science, The Pennsylvania State University, University Park, PA, United States

**Keywords:** climate change, crop wild relatives, endangered wild species, environmental stresses, nutritional development, molecular strategies, genetic variation, omics approach

The demand of food is expected to increase significantly in the coming decades with growing population and shifting dietary patterns (Tilman et al., 2011). Crop yield is largely affected by various factors including biotic and abiotic stresses, reduced biodiversity, degraded soil, and the changing climate (Lobell and Gourdji, 2012; Islam et al., 2016; Zhu et al., 2022). Plant genetic resources including crop wild relatives (CWRs), landraces and underutilized crop species are a significant source of traits of agronomic interest (Khan et al., 2021; Renzi et al., 2022). Conserving and utilizing such genetic resources may be key for the development of climate resilient crop varieties and to ensure global food and nutrition security (**Figure 1**). The conservation of plant genetic resources and the availability of their omics data is critical for the improvement of crop varieties using advanced molecular breeding including genome editing (Islam, 2019; Salgotra and Chauhan, 2023). For the efficient utilization, genetic resources should be comprehensively examined, however, there has been a dearth in the assessment of their heritable traits and full characterization (**Figure 1**). Moreover, more studies are required on the assessment of genetic variability, level of tolerance against individual and combined biotic and abiotic stresses, yield performance and nutritional profile of wild plant genetic resources (Panwar et al., 2022; Pandey et al., 2023). Therefore, this Research Topic aimed to compile research updates on successful monitoring and utilization of wild plant genetic resources for modern crop improvement.

**Figure 1:**
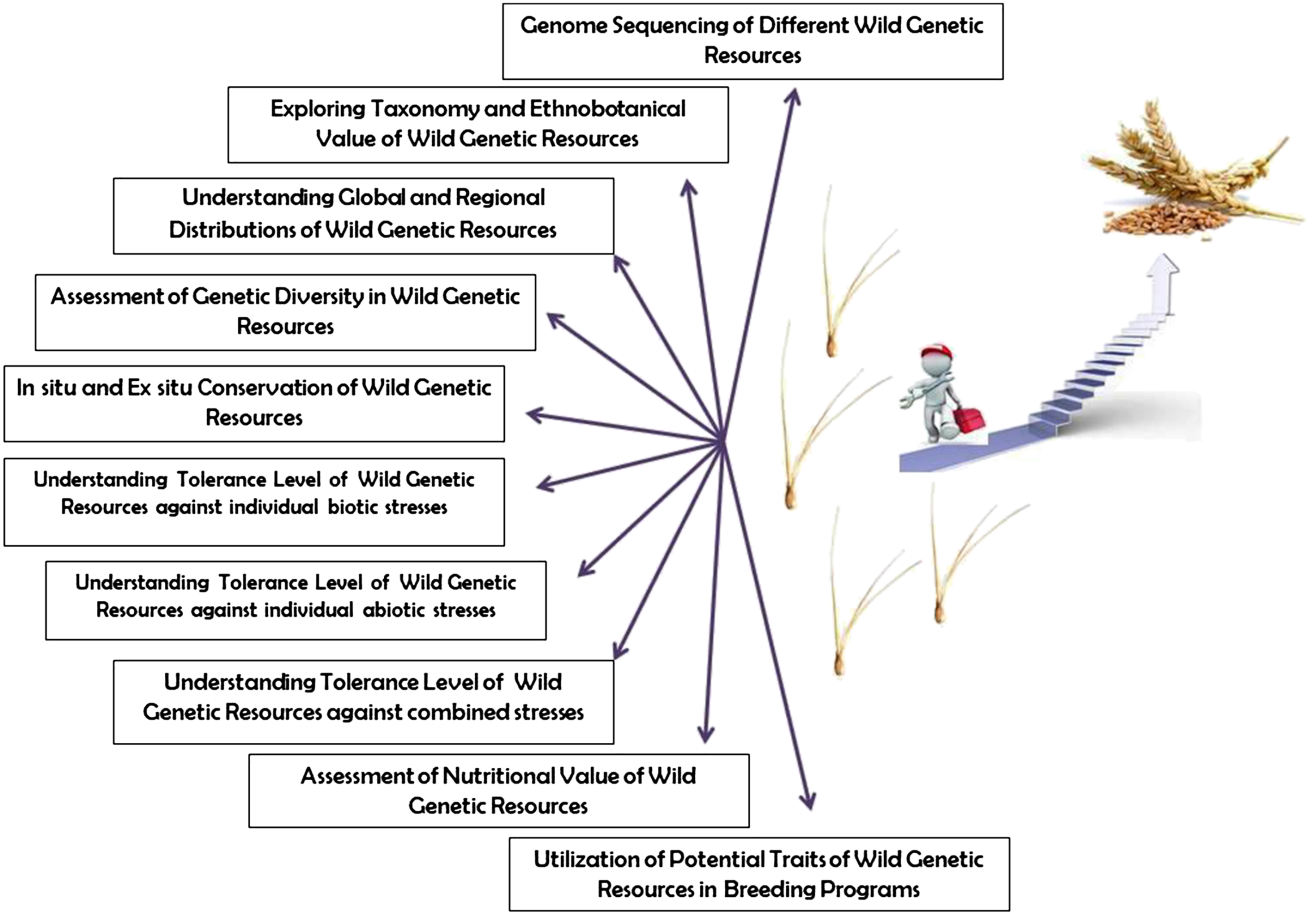
Outline of different aspects of wild genetic resources that need to be investigated further for the development of modern cultivars.

The research findings and critical reviews described in eight articles included in this Research Topic are briefly summarized and discussed in the following sections.

## 1 Steps toward efficient conservation of CWRs

Being an important source of traits for biotic and abiotic stress tolerance, and nutrients, crop wild relatives (CWRs) can largely contribute to the improvement of crops (Kahraman et al., 2017; Rajpal et al., 2023). However, these natural genetic resources are under continuous threat due to global climate change and anthropogenic activities. Therefore, conservation of these valuable genetic resources must be prioritized. Brilhante et al. catalogued the diversity of CWRs of genus *Vigna* in Mozambique and provided conservation strategies for preferred targets and regions. This inclusive dataset containing information on species diversity, dissemination, and threats will facilitate the sustainable usage of *Vigna* CWRs in crop improvement. They suggested that climatic fluctuations and changes in agricultural practices in that region destruct natural habitats and threaten the existence of wild populations. Chen et al. explored the influence of land use change in China from 2001 to 2019 on the survival rate of three wild rice species (*Oryza rufipogon, O. officinalis*, and *O. granulata*) employing satellite-based Earth observations. Although the land use change had a suppressive effect on the population of three wild rice species, it has been suggested that vegetation surrounding the wild population acts as a biological barrier and protects the plants from the destruction due to land use change. The findings of their study emphasize the need of a modified conservation strategy for wild species of rice. Other than this, the loss of wild species because of land use change emphasizes the *in situ* conservation of wild relatives (Thingnam et al., 2023). However, genetic characterization of individual samples of such large populations is difficult due to requirement for the large resources. Thus, tolerance level of large populations of CWRs towards different stress conditions at difficult sites can be easily identified using predictive characterization techniques based on ecogeographic information. Civantos-Gomez et al. employed a machine learning-based predictive strategy to characterize the resistance toward rust disease caused by fungus *Uromyces viciae-fabae* in CWR populations of lentils in the Mediterranean basin. They concluded that rust resistant CWR populations are suitable candidates for *in situ* conservation, and specific environmental conditions have a role in developing resistance in them. Davis et al. provided a contemporary review summarizing taxonomy, conservation, and potential traits including agronomic performance and biotic/abiotic stress tolerance of four indigenous species of coffee in Uganda. This review not only identified that two of the studied coffee species are at the risk of extinction but also suggested to prioritize their conservation in Uganda.

## 2 Utilization of genetic resources to maintain diversity

An efficient way of utilizing CWRs is to improve the strategies of their selection based on phenotypic and genomic indices. Fenstemaker et al. utilized phenotypic, genomic, and combined strategies to select water-deficit stress tolerant lines developed from the crosses of tolerant wild tomato relative *Solanum galapagense* accession LA1141 and susceptible *Solanum lycopersicum* L. OH8245. Thermal images showed a greater phenotypic variance for canopy temperature trait in the progenies and quantitative trait loci (QTLs) contributing to water deficit tolerance which were mapped in LA1141. These findings suggest the opportunity to introgress water deficit tolerance trait from wild relatives to modern tomato cultivars. Moreover, understanding the material developed from the breeding of CWRs can facilitate its utilization for crop improvement. Sequencing of whole genomes and transcriptomes of wild genetic resources will be advantageous in this direction (Brozynska et al., 2016; Pandey et al., 2022; Khan et al., 2023). Jackfruit (*Artocarpus heterophyllus* Lam, Moraceae family) has attracted the attention of food experts and technologists due to its nutritional health benefits. However, it is an underutilized and less explored tropical fruit crop, but a highly potential source of food and nutritional security. Islam et al. for the first time sequenced the whole genome of a year-round fruiting and high yielding jackfruit variety, BARI Kanthal-3, which was developed from a wild accession. Bioinformatics analysis of the sequence data identified the distribution of a large collection of nucleotide variation across the genome of jackfruit that can be used to identify new functional genes and their regulatory activities specific to BARI Kanthal-3. They also demonstrated that BARI Kanthal-3 has a higher number of genes related to flowering time. Their findings not only facilitate marker development for different traits in jackfruit crop to be utilized in breeding programs, but also increase the chances of their utilization to ensuring food supply by understanding their evolution and domestication process.

Reproductive isolation limits the utilization of wild species for the improvement of cultivated forms. Thus, other existing forms that are closer to widely utilized cultivated genotypes and that possess potential stress tolerance and yield related traits should be preferred for crop improvement. The Waxy (Wx) gene is found to be responsible for waxy trait in waxy rice that is high-quality rice with less than 2% of apparent amylose content (AAC) of the starch. Fu et al. developed wx mutants of rice varieties employing CRISPR-Cas9 gene-editing system that showed significant decrease in AAC. However, AAC content of rice genotypes with low initial AAC was further decreased on mutation of wx genes and thus, preference can be given to such genotypes in breeding programs. Li et al. studied a semi-domesticated rice called weedy rice (*Oryza sativa* f. *spontanea*) for the development of early heading trait in japonica rice. Four genes, two major (*Hd1* and *OsCCT22*) and two minor (*Dth7* and *Hd16*) were found to be regulating the early heading trait of weedy rice.

The articles published in this Research Topic address different aspects of the conservation and utilization of wild plant genetic resources, however, a consistent research effort is needed for the efficient conservation and utilization of the invaluable patrimony of wild plant genetic resources (**Figure 1**).

## Author contributions

MKK conceived the idea of the Research Topic, wrote the editorial, sketched the figure and acted as editor of some of the manuscripts of the Research Topic. TI, SG, and FG, acted as editor of some of the manuscripts of the Research Topic. TI, SG, FG along with MKK edited and reviewed this editorial. All authors contributed to the article and approved the submitted version.

## References

[B1] Brozynska M. Furtado A. Henry R. J. (2016). Genomics of crop wild relatives: expanding the gene pool for crop improvement. Plant Biotechnol. J. 14, 1070–1085. doi: 10.1111/pbi.12454 26311018 PMC11389173

[B2] Islam T. (2019). CRISPR-cas technology in modifying food crops. CAB Rev. 14, 1–16. doi: 10.1079/PAVSNNR201914050

[B3] Islam M. T. Croll D. Gladieux P. Soanes D.M. Persoons A. Bhattacharjee P.. (2016). Emergence of wheat blast in Bangladesh was caused by a south American lineage of *Magnaporthe oryzae* . BMC Biol. 14, 84. doi: 10.1186/s12915-016-0309-7 27716181 PMC5047043

[B4] Kahraman A. Pandey A. Khan M. K. Lindsay D. Moenga S. Vance L.. (2017). Distinct subgroups of *Cicer echinospermum* are associated with hybrid sterility and breakdown in interspecific crosses with cultivated chickpea. Crop Sci. 57, 3101–3111. doi: 10.2135/cropsci2017.06.0335

[B5] Khan M. K. Pandey A. Hamurcu M. Avsaroglu Z. Z. Ozbek M. Omay A. H.. (2021). Variability in physiological traits reveals boron toxicity tolerance in aegilops species. Front. Plant Sci. 12. doi: 10.3389/fpls.2021.736614 PMC8585849 34777419

[B6] Khan M. K. Pandey A. Hamurcu M. Rajpal V. R. Vyhnanek T. Topal A.. (2023). Insight into the boron toxicity stress-responsive genes in boron-tolerant triticum dicoccum shoots using RNA sequencing. Agronomy 13, 631. doi: 10.3390/agronomy13030631

[B7] Lobell D. B. Gourdji S. M. (2012). The influence of climate change on global crop productivity. Plant Physiol. 160, 1686–1697. doi: 10.1104/pp.112.208298 23054565 PMC3510102

[B8] Pandey A. Khan M. K. Athar T. Hamurcu M. Germ M. Gezgin S. (2023). “Chapter 17 - combined abiotic stresses in wheat species,” in Abiotic stresses in wheat. Eds. Khan M. K. Pandey A. Hamurcu M. Gupta O. P. Gezgin S. (San Diego, CA, United States: Academic Press), 273–282.

[B9] Pandey A. Khan M. K. Hamurcu M. Brestic M. Topal A. Gezgin S. (2022). Insight into the root transcriptome of a boron-tolerant triticum zhukovskyi genotype grown under boron toxicity. Agronomy 12, 2421. doi: 10.3390/agronomy12102421

[B10] Panwar R. Chaudhry B. Kumar D. Prakash G. Khan M. K. Pandey A.. (2022). Harnessing stress-tolerant wild bananas for crop improvement. Crop Pasture Sci. doi: 10.1071/CP22294

[B11] Rajpal V. R. Singh A. Kathpalia R. Thakur R. K. Khan M. K. Pandey A.. (2023). The prospects of gene introgression from crop wild relatives into cultivated lentil for climate change mitigation. Front. Plant Sci. 14. doi: 10.3389/fpls.2023.1127239 PMC10044020 36998696

[B12] Renzi J. P. Coyne C. J. Berger J. Von Wettberg E. Nelson M. Ureta S.. (2022). How could the use of crop wild relatives in breeding increase the adaptation of crops to marginal environments? Front. Plant Sci. 13, 886162. doi: 10.3389/fpls.2022.886162 35783966 PMC9243378

[B13] Salgotra R. K. Chauhan B. S. (2023). Genetic diversity, conservation, and utilization of plant genetic resources. Genes 14, 174. doi: 10.3390/genes14010174 36672915 PMC9859222

[B14] Thingnam S. S. Lourembam D. S. Tongbram P. S. Lokya V. Tiwari S. Khan M. K.. (2023). A perspective review on understanding drought stress tolerance in wild banana genetic resources of northeast India. Genes 14, 370. doi: 10.3390/genes14020370 36833297 PMC9957078

[B15] Tilman D. Balzer C. Hill J. Befort B. L. (2011). Global food demand and the sustainable intensification of agriculture. Proc. Natl. Acad. Sci. U.S.A. 108, 20260–20264. doi: 10.1073/pnas.1116437108 22106295 PMC3250154

[B16] Zhu P. Burney J. Chang J. Jin Z. Mueller N.D. Xin Q.. (2022). Warming reduces global agricultural production by decreasing cropping frequency and yields. Nat. Clim. Change 12, 1016–1023. doi: 10.1038/s41558-022-01492-5

